# An integrated bioinformatic investigation of mitochondrial energy metabolism genes in colon adenocarcinoma followed by preliminary validation of CPT2 in tumor immune infiltration

**DOI:** 10.3389/fimmu.2022.959967

**Published:** 2022-09-13

**Authors:** Zichao Cao, Jianwei Lin, Gang Fu, Lingshan Niu, Zheyu Yang, Wei Cai

**Affiliations:** Department of General Surgery, Ruijin Hospital, Shanghai Jiao Tong University School of Medicine, Shanghai, China

**Keywords:** mitochondrion, energy metabolism, colon adenocarcinoma, immune, CPT2

## Abstract

**Background:**

The prognosis for colon adenocarcinoma (COAD) today remains poor. Changes in mitochondria-related genes and metabolic reprogramming are related to tumor growth, metastasis, and immune evasion and are key factors in tumor genesis and development.

**Methods:**

TCGA database was used to analyze the differentially expressed mitochondrial energy metabolism pathway-related genes (MMRGs) in COAD patients, and the mutation of MMRG in tumor cells, the biological processes involved, and the correlation with tumor immunity were also analyzed. Then, MMRG and MMRG-related genes were used to divide COAD patients into different subtypes, and immunocorrelation analysis and survival analysis were performed. Finally, univariate regression analysis and LASSO regression analysis were used to construct a prognostic risk model for COAD patients, which was verified by the GEO database and evaluated by Kaplan–Meier (K-M) and receiver operating characteristic (ROC) curves, and the correlation between the risk model and immunity and clinical subtypes based on MMRG was analyzed.

**Results:**

In this study, the MMRG patterns and tumor immune microenvironment characteristics in COAD patients were systematically evaluated by clustering the expression of 188 MMRGs. We identified two subtypes of COAD with different clinical and immunological characteristics. Eight of the 28 differentially expressed MMRG genes were used to construct risk scores. ROC and K-M curves suggested that the risk model could well predict the prognosis of COAD patients, and the risk model was related to immune cell infiltration and immune function.

**Conclusions:**

The two COAD subtypes identified by MMRG are helpful for the clinical differentiation of patients with different prognoses and tumor progressions, and the risk score can assist the clinical evaluation of patient prognosis. Our results suggest that CPT2 contributes to the recruitment and regulation of neutrophils in COAD. CPT2 may act as a valuable biomarker for COAD immunotherapy.

## Introduction

High incidence and mortality rates have made colorectal cancer a major public health problem (1, 2). Colon adenocarcinoma (COAD) is the most common pathological type of colorectal cancer (3). Recently, the onset age of COAD has been becoming younger and more aggressive, which makes the treatment of COAD confronted with more severe challenges (4). Metabolic reprogramming in COAD can drive tumor progression and influence tumor metastasis (5). Many tumors maintain survival in an environment of nutrient scarcity and oxidative stress by enhancing glycolysis and promoting changes in mitochondrial metabolism (6, 7). Therefore, cellular metabolic pathways, especially mitochondrial metabolism, not only affect the occurrence and development of colorectal cancer but also are potential targets for tumor therapy.

The mitochondria provide most of the biological energy for the activities of organisms and are important production sites of catabolism of organisms, as well as key regulatory factors of cell proliferation and apoptosis (8). Mitochondrial dysfunction is closely related to many congenital diseases, inflammation, and tumors (9, 10). In tumors, mitochondria are key factors in tumor progression. They not only provide ATP for tumors but also produce reactive oxygen species that promote the accumulation of oncogenic DNA (11, 12). In terms of tumor metastasis, mitochondrial metabolites can promote the epithelial–mesenchymal transformation (EMT) of tumor cells, thus enhancing the invasion ability of cancer cells (13–15). In the aspect of malignant transformation, mitochondrial outer membrane permeability or mitochondrial permeability transformation is necessary for the early survival of cancer cells (16, 17). In the aspect of tumor immunity, mitochondria participate not only in the activation of inflammasome but also in the differentiation of memory immune cells and macrophages and the activation of antitumor activity (18–21). This indicates that mitochondria play an important role in tumor, and mitochondria, as the production site of energy metabolism, affect the process of mitochondrial energy metabolism by changing mitochondrial genes and metabolic patterns. Therefore, abnormal mitochondrial energy metabolism pathway-related genes (MMRGs) may lead to abnormal energy production, thus affecting the occurrence and development of COAD.

In this study, we systematically analyzed 188 MMRGs in colorectal adenocarcinoma, including their expression levels, mutations, participating biological functions, and correlation with the tumor immune microenvironment using The Cancer Genome Atlas (TCGA) database. In addition, the 188 MMRGs were used to identify two COAD subtypes with different clinical and immune characteristics and were used to construct a prognostic risk score model which was verified by the Gene Expression Omnibus (GEO) database. COAD subtypes differentiated based on MMRGs can distinguish patients with different clinical prognoses effectively, and the risk model can well predict the prognosis of patients with COAD. Furthermore, we initially validated our findings in clinical specimens and explored the immunocorrelation of the identified member CPT2 in colon cancer-derived cell lines.

## Materials and methods

### Data collection

The mRNA expression data, mutant maf data, and clinical information of COAD patients were downloaded from TCGA database (https://portal.gdc.cancer.gov/repository) and GEO database (http://www.ncbi.nlm.nih.gov/geo/). These data contain 437 samples (39 normal tissue, 398 COAD tissues) in TCGA and 579 cases in GSE39582. The GEO samples were analyzed by the Affymetrix Human Genome U133 Plus 2.0 Array platform. All cases from TCGA or GEO database that miss the information were excluded from analysis. The clinical characters of patients (age, gender, stage, T stage, N stage, M stage) were recorded. Unknown clinical characteristics were deleted.

### Differential expression gene and gene mutation analysis

The summary and collection of complete MMRGs refer to the research of Ye et al. (22). The R-package limma was used to analyze MMRGs that were differentially expressed in normal and tumor tissues and to map the genes used to build risk models in the volcano map. R package maftools was used to visually analyze the mutation frequency of patients’ mutated genes. FDR value ≤0.05 and | log2 fold change | ≥1 were used as cutoff values to analyze differentially expressed genes.

### Gene set enrichment analysis

GSEA software (version 3.0) was used to analyze the gene set enrichment, and the samples can be divided into normal tissue and tumor tissue groups. c5.go.bp.v7.4.symbols.gmt was used as the database to evaluate the biological processes involved in normal and tumor tissues of the 188 MMRGs in COAD patients. Based on gene expression profile and phenotypic grouping, the minimum gene set was set as 5, and the maximum gene set was set as 5,000. The p value <0.05 was considered statistically significant.

### Protein–protein interaction network

The STRING database (https://string-db.org/) is set for searching online for known protein interoperability relationships. We used this database to analyze and predict the functional relationship among MMRGs, and the cytoHubba plugin in the software of Cytoscape (version 3.8.1) was used to show the correlation between MMRG in our risk model and other MMRGs.

### Correlation analysis of immune cell infiltration and immune function

The “GSVA” R package was utilized to conduct the ssGSEA to calculate the scores of infiltrating immune cells and to evaluate the activity of immune-related pathways. Then, the correlation between the expression level of differentially expressed MMRGs and immune cells as well as immune function was analyzed. Meanwhile, in the subsequent tumor classification and tumor risk model, the differences in immune cell infiltration and immune function between different tumor types and different risk groups was analyzed. We reexamined the association between *CPT2* expression and common immune cell abundance using the Tumor Immune Estimation Resource (TIMER 2.0).

### Unsupervised clustering for MMRG

Unsupervised clustering methods were used to identify different MMRG patterns and classify patients for further analysis. A total of 188 MMRG genes were used to conduct the unsupervised clustering. A consensus clustering algorithm was performed using the R package ConsensusClusterPlus.

### Risk model construction

The correlation between 28 differentially expressed genes and prognosis was analyzed by univariate Cox regression. In order not to omit genes that can be used to build the model, we set the p value to 0.2. Then, LASSO cox regression analysis was performed using the R package to construct the risk model. Each COAD patient risk score was calculated by this model and used to divide patients into two groups (low-risk and high-risk groups) by the value which was determined by the software of X-tile (23). The receiver operating characteristic (ROC) and Kaplan–Meier (K-M) curves were used to evaluate the prognostic ability of the risk model. GSE39582 was regarded as the validation set to verify the predictive ability of the prognostic risk model based on TCGA database.

### Survival prognosis and clinical stage analysis

The overall survival (OS) survival map data and pathological stage plot for MMRGs in colorectal adenocarcinoma in TCGA database were obtained *via* the GEPIA2 online website.

### Human neutrophil isolation

An alternative method for the isolation of neutrophils using a discontinuous density gradient composed of two solutions of a radiopaque medium of differential density (Histopaque-1077 and -1119) is commercially available from Sigma. Following the manufacturer’s procedure, neutrophils can be enriched from human peripheral blood. In the next step, the erythrocytes were lysed with a hemolytic solution for 10′ (93.00 g/l NH4Cl, 10.00 g/l KHCO_3_, and 0.40 g/l EDTA, pH 7.2). The neutrophil pellet, free of erythrocyte debris after a 300 g centrifugation of 10′, was washed two times with HBSS and centrifuged twice at 300 g for 10′ each time. A new neutrophil pellet was obtained after removing the HBSS washing solution.

### Cell line culture

Human CRC cell line RKO was acquired from the American Type Culture Collection (ATCC, Manassas, VA, USA) and was routinely cultured in Dulbecco’s modified Eagle’s medium (DMEM; Meilun, China) supplemented with 10% fetal bovine serum (FBS; Gibco) and 100 U/ml penicillin and 100 μg/ml streptomycin (Meilun, China).

### Transient transfection

Small interfering RNA for CP2 (si-CPT2, 150 nM) was transfected into RKO and SW480 to knock down CPT2. si-CPT2 was designed and produced by GeneChem (Shanghai, China). Cell transfection was performed by using Lipofectamine^®^ 2000 (Invitrogen) at indicated times.

### Validation in a tissue microarray using immunohistochemistry

A total of 61 patients with colon cancer were pathologically diagnosed at Ruijin Hospital Affiliated to Shanghai Jiaotong University from October 2012 to January 2016. The patients were informed of the study and signed informed consent forms. Furthermore, the study was approved by the ethics committee of Ruijin Hospital. Tissue microarray analysis was performed as described previously, immunostained with primary antibodies against CPT2 (ProteinTech, 26555-1-AP) and MPO (ProteinTech, 66177-1-Ig) overnight at approximately 4°C, and subsequently incubated with a goat anti-rabbit secondary antibody (Servicebio Technology, Wuhan, China) for 30 min at approximately 20°C. Immunohistochemistry (IHC) results were scored as previously described. CPT2 was scored on a sliding scale according to the percentage of positive cells (0 = 0%, 1 = 1%–20%, 2 = 21%–50%, 3 = 51%–80%, 4 = 81%–100%) and the staining intensity (0 = negative, 1 = weak, 2 = moderate, 3 = strong). The two scores were multiplied to generate an immunoreactive score (IRS) ranging from 0 to 12, and neutrophil infiltration was scored according to the percentage of infiltration (%).

### RNA isolation and real-time PCR

Real-time reverse transcription–PCR was performed as described previously. The primers used for quantitative PCR were as follows: CPT2, 5′-CATACAAGCTACATTTCGGGACC-3′ forward and 5′-AGCCCGGAGTGTCTTCAGAA-3′ reverse GAPDH, 5′-GAAATCCCAT CACCATCTTCCAGG-3′ forward and 5′-GAGCCCCAGCCTTCTCCATG-3′ reverse. All PCR experiments were performed in triplicate.

### Transwell migration assay

Neutrophil migration was measured in a Transwell chamber (3 μm, Corning Costar, USA). Neutrophils (5 × 10^5^ cells) were added to the upper chamber, and the supernatant of CRC cells was added to the lower chamber. After 2 h of incubation, the number of neutrophils located in the lower chamber was counted.

### Flow cytometry

The apoptosis rate of neutrophil cells was determined by flow cytometry using a dead cell apoptosis kit with Annexin V and 7-AAD (BD Biosciences). Briefly, 200 μl binding buffer containing 5 μl Annexin V and 7 μl 7AAD was added to the samples for 1 h in the dark. The stained cells were analyzed using FACSCalibur™ flow cytometry (BD Biosciences, San Jose, CA, USA) and CellQuest software.

### Statistical analysis

R version 4.0.5 and Perl version 5.28 were used to perform statistical analysis. Excel office 2019 was used to organize data from TCGA and GEO databases. Except that the p-value <0.2 was set as the condition for screening prognostic genes in univariate Cox regression analysis, the p-value <0.05 was used as the significant condition for others without special explanation.

## Results

### Differentially expressed genes

**Figure 1:**
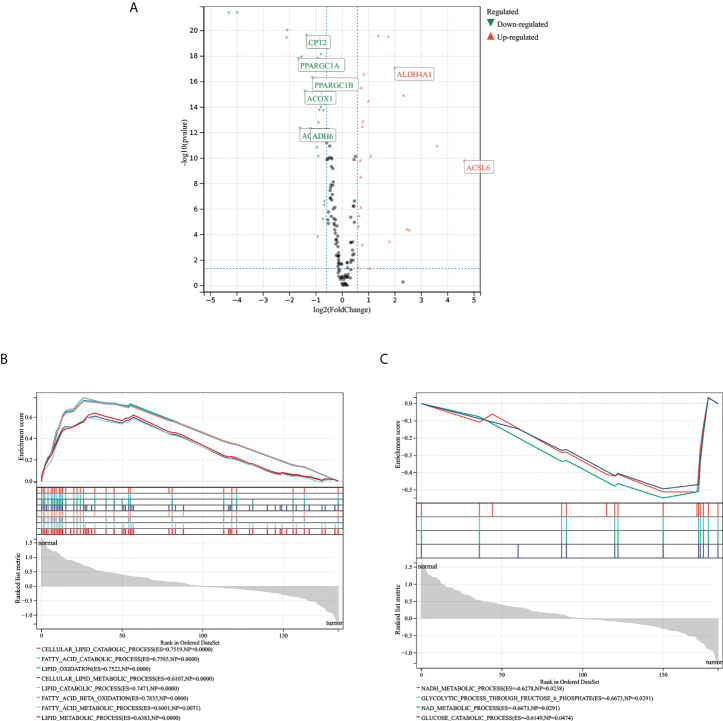
Expression and biological processes of the MMRG gene. **(A)** Volcanic map of MMRG gene expression in COAD; green represents downregulation in tumors, red represents upregulation in tumors. **(B, C)** The biological processes of MMRG, which were activated in normal tissue on the left and tumor tissue on the right. MMRGs, mitochondrial energy metabolism pathway-related genes; COAD, colon adenocarcinoma.

A total of 28 MMRGs were differentially expressed in normal and tumor tissues in patients with COAD. Of these, 16 genes (ADH1B, ADH1A, ADH1C, ACADS, PPARGC1A, ACADL, ACAA2, ACOX1, CPT2, ECI2, ADH6, ACADM, ACAT1, EHHADH, PPARGC1B, CPT1A) were downregulated in tumors and 12 (PPAN, ACSBG2, ACSL4, PPA1, PPAT, ALDH7A1P1, ALDH4A1, CYP4A22-AS1, ACSL3-AS1, PPATP1, ALDH3B2, ACSL6) were upregulated in tumors. The volcano diagram in **Figure 1A** visually shows the expression of differentially expressed genes.

### Gene function, mutation, and PPI analysis of MMRGs

**Figure 2:**
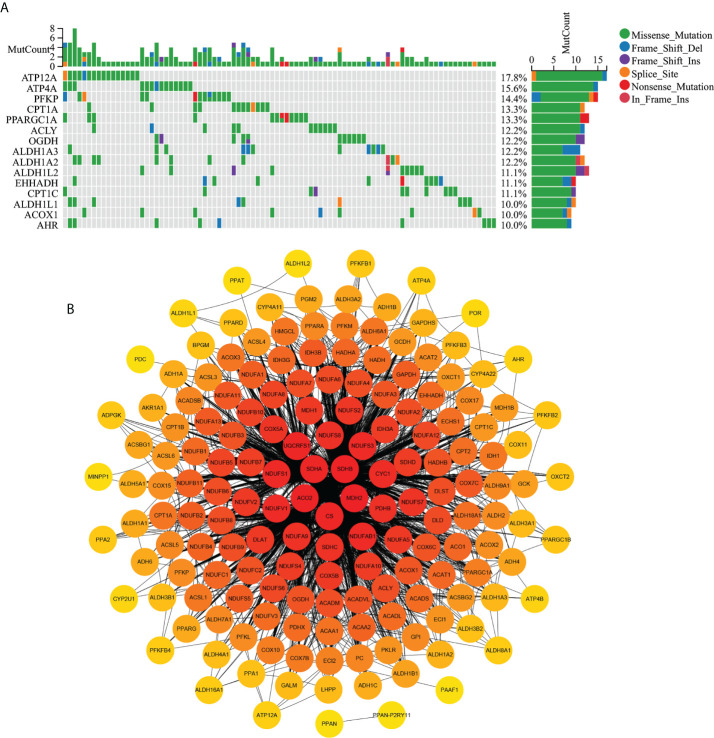
Map of MMRG gene mutation and protein interaction in COAD. **(A)** The first 15 MMRGs mutated in COAD. **(B)** MMRG protein interaction diagram; the darker the red, the more critical the protein is.

Results of biological process analysis (**Figures 1B, C**) showed that 188 MMRGs were mainly involved in lipid metabolism in normal tissues and were mainly involved in ATP energy generation-related pathways (such as oxidative phosphorylation) in tumor tissues. These results suggest changes in mitochondrial energy metabolism in COAD. In **Figure 2A**, gene mutation analysis frequency showed that the MMRG mutation frequency was high in COAD, with the highest frequency reaching 17.8%. The mutation frequency of the top 15 genes with the highest mutation frequency was not less than 10%. The protein–protein interaction (PPI) network shows (**Figure 2B**) that MMRGs have a wide range of protein interactions, and the proteins of MMRGs form a complex network that jointly participates in the process of mitochondrial energy metabolism, thus affecting the transformation of energy metabolism model in tumor and promoting tumor progression.

### Immunocorrelation of MMRG in colorectal adenocarcinoma

**Figure 3:**
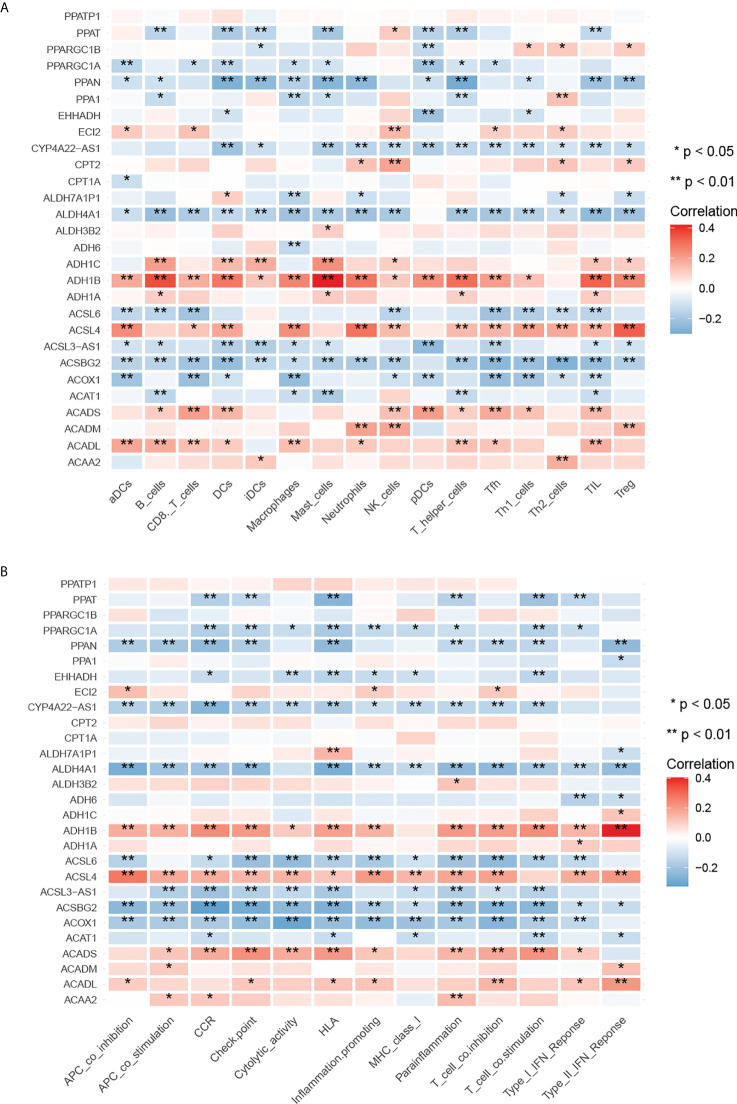
Correlation between differentially expressed MMRGs and immune characteristics. **(A)** Correlation between differentially expressed MMRGs and tumor immune cell infiltration; red is positive, blue is negative. **(B)** Correlation between differentially expressed MMRGs and tumor immune function, red is positive, blue is negative. *p < 0.05; **p < 0.01.

To understand the value of MMRGs in tumor immunity, we analyzed the correlation of 28 differentially expressed MMRGs with tumor immune cell infiltration and immune function. The analysis results indicated (**Figures 3A, B**) that the expression levels of most MMRGs were correlated with various immune cell infiltrates in the tumor immune microenvironment, as well as with various immune function processes. These results suggested that MMRGs may be involved in the process of tumor immune invasion.

### Clinical typing by MMRGs in TCGA COAD patients

**Figure 4:**
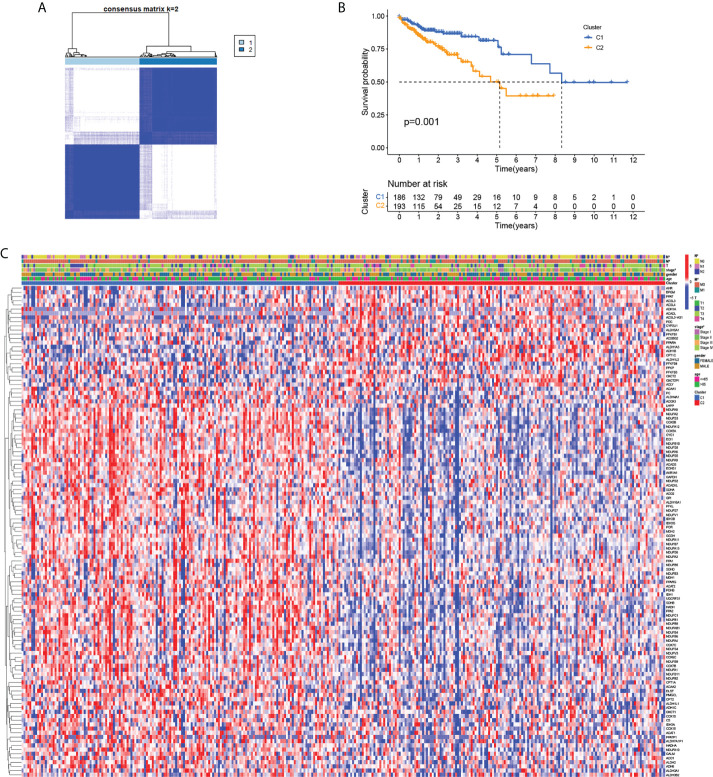
Tumor classification based on the MMRGs. **(A)** COAD patients were grouped into two clusters according to the consensus clustering matrix (k = 2). **(B)** Kaplan–Meier curves for the two clusters. **(C)** Heatmap for the expression of MMRGs and clinical features between two clusters; red represents high expression, and blue represents low expression.

In order to understand the relationship between MMRG and the COAD subtype, consensus clustering analysis was used to analyze the patients from TCGA database. As the clustering variable (k) increased (from 2 to 10), intragroup connections were the highest and intergroup connections were the lowest when k = 2 (**Figure 4A**), indicating that the COAD patients could be well divided into two subtypes. The results of survival analysis suggested (**Figure 4B**) that there were differences in the survival of the two COAD subtypes based on 188 MMRG genes. The prognosis of subtype C2 is worse than that of C1. A clinically relevant heat map (**Figure 4C**) showed that the expression of most MMRGs decreased in the C2 subtype, and there were differences in N, M, and TNM staging between the two COAD subtypes. These results suggest that COAD typing based on MMRG can well distinguish the prognosis of COAD patients, and this typing reflects the differences in lymph node metastasis, distant metastasis, and TNM stage of patients.

### Differential immune characteristics of MMRG patterns

**Figure 5:**
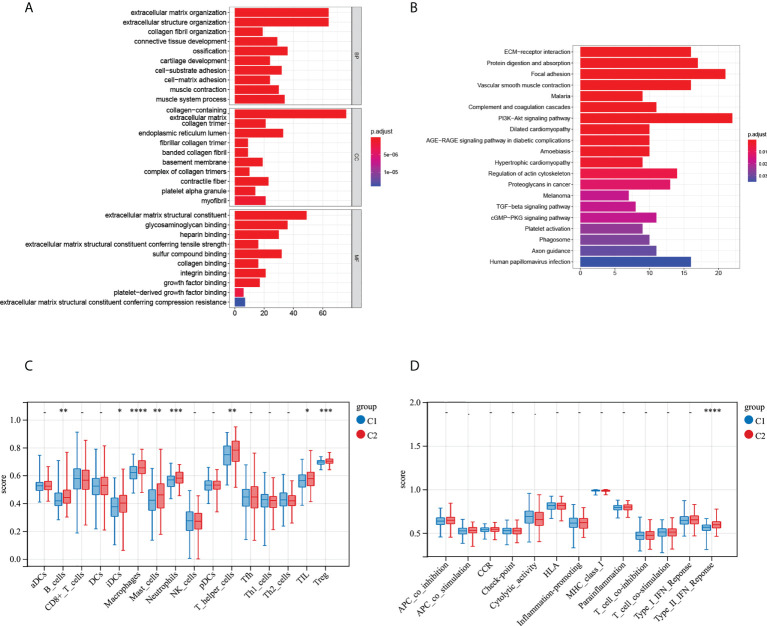
Differential immune characteristics of MMRG patterns C1 and C2. **(A)** GO bar graph for genes in BP, CC, and MF. **(B)** Bubble graph of the top five KEGG pathways with the most enriched genes; the vertical axis refers to the names of the pathway, and the horizontal axis refers to the number of genes. **(C)** Relative infiltration of 16 types of immune cells in MMRG clusters C1 and C2. **(D)** Relative enrichment score of 13 immune-related functions in MMRG clusters C1 and C2. GO, Gene Ontology; BP, biological process; CC, cellular component; MF, molecular function; KEGG, Kyoto Encyclopedia of Genes and Genomes. *p < 0.05; **p < 0.01; ***p < 0.001; ****p < 0.0001.

In order to understand the functions and signaling pathways of differentially expressed genes among COAD subtypes, 633 differentially expressed genes (p < 0.05, |log2 fold change| ≥1) among COAD subtypes were analyzed. Gene Ontology (GO) analysis results showed (**Figure 5A**) that genes differentially expressed between the two subtypes were involved in extracellular matrix organization, extracellular structure organization, collagen fibril organization and other biological processes (BP), collagen-containing extracellular matrix, collagen trimer and other cellular component (CC), extracellular matrix structural constituent, glycosaminoglycan binding, and other molecular function (MF). The Kyoto Encyclopedia of Genes and Genomes (KEGG) pathway enrichment analysis is exhibited in **Figure 5B**. **Figures 5C, D** showed differences in immune cell infiltration and immune function involvement among subtypes, and the results showed that B cells, immature dendritic cells (iDCs), macrophages, mast cells, neutrophils, T helper cells, tumor infiltrating lymphocytes (TIL), and Treg cells in C2 showed higher levels of infiltration compared with C1. In addition, type C2 showed a higher type II IFN response. These results suggest that there are differences in immune infiltration among patients of this classification, which may provide guidance for immunotherapy.

### The clinical and transcriptomic characteristics of MMRG-related gene clusters

**Figure 6:**
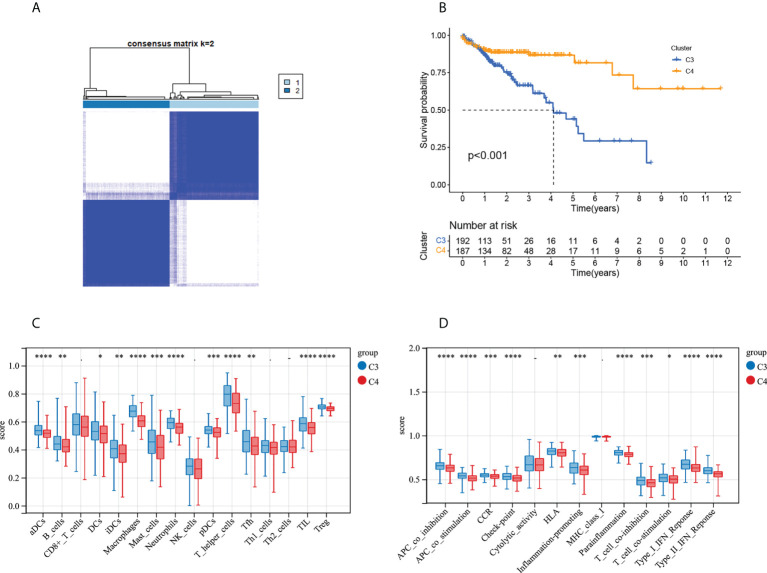
Tumor classification based on the MMRG-related gene. **(A)** COAD patients were grouped into two clusters according to the consensus clustering matrix (k = 2). **(B)** Kaplan–Meier curves for the two clusters. **(C)** Relative infiltration of 16 types of immune cells in MMRG-related gene clusters C3 and C4. **(D)** Relative enrichment score of 13 immune-related functions in MMRG-related gene clusters C3 and C4. *p < 0.05; **p < 0.01; ***p < 0.001; ****p < 0.0001.

The heatmap of the 633 gene expressions is shown in **Supplementary Figure 1**. Most of the genes were upregulated in C2 type. According to the above analysis results, these genes are mainly responsible for extracellular matrix remodeling and biological processes. In order to further explore the heterogeneity of COAD under different MMRG modes, unsupervised cluster analysis was performed on patients according to the 633 differentially expressed genes, and COAD patients were further divided into two subtypes (**Figure 6A**). The results of survival analysis (**Figure 6B**) showed that there were survival differences between two subtypes, and the prognosis of the C3 subtype was worse than that of the C4 subtype. The difference in immune cell infiltration and immune function between C3 and C4 subtypes is shown in **Figures 6C, D**.

### Risk model construction

Univariate cox regression was used to analyze the correlation between 28 differentially expressed MMRGs and their prognosis. In order not to omit genes that could be used to build risk models, the p value was set to 0.2, and a total of 10 genes were screened out (**Supplementary Figure 2A**). LASSO Cox regression analysis was then used to further screen the genes used to build the model and build the risk model. A total of eight genes were used to build the risk model (**Supplementary Figure 2B**). The risk scoring formula is as follows:


$$\begin{array}{l} riskScore\;=\;ACOX1*-0.3749+CPT2*-0.4624+ADH6*\\ -0.0597+\;PPARGC1A*-0.1632+ACSL6*\\ -0.0865+ACADL*1.2387+\;PPARGC1B*\\ -0.2247+\;ALDH4A1*-0.1972 \end{array}$$

**Figure 7:**
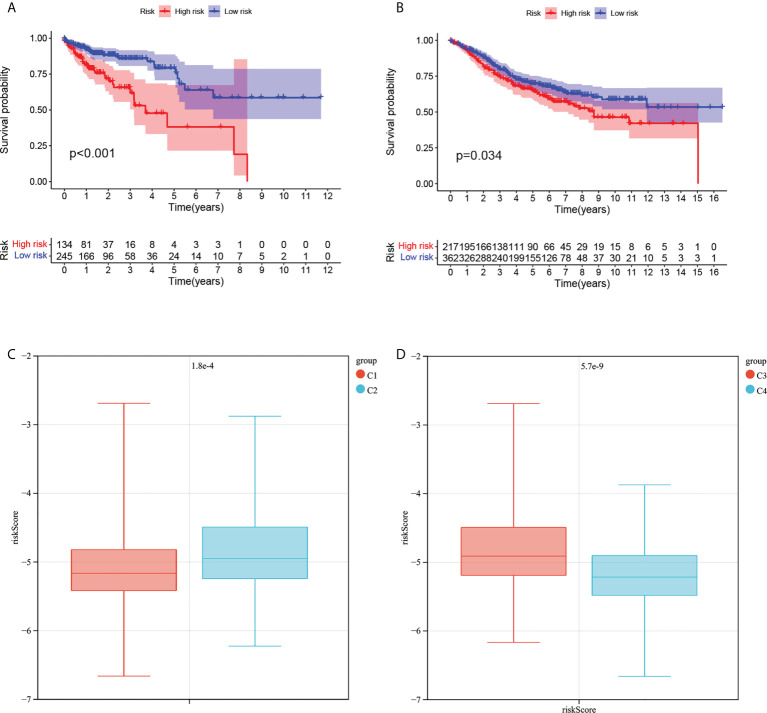
Survival analysis of risk models and correlation with COAD subtypes. **(A)** Analysis of survival differences between high-risk and low-risk COAD patients in TCGA database. **(B)** Analysis of survival differences between high-risk and low-risk COAD patients in GSE39582. **(C)** The correlation of risk score with the MMRG pattern. **(D)** The correlation of risk score with the MMRG-related gene pattern.

The risk score of COAD patients in TCGA and GEO databases was calculated according to the risk model, and the patients were divided into high-risk and low-risk groups according to the cutoff value of -4.88 determined by X-tile. The survival analysis showed that the survival of patients in the high-risk group was worse than that in the low-risk group (**Figures 7A, B**). The ROC and principal component analysis (PCA) analysis results of the risk model constructed by using TCGA data are shown in **Supplementary Figures 2C, D**, suggesting that the risk model could predict the prognosis of COAD patients and distinguish COAD patients with different risks well. Analysis of the correlation between COAD typing and risk model constructed by MMRG and MMRG-related genes showed that the risk score of the C2 and C3 COAD subtypes was higher than that of C1 and C4, which was consistent with the poor prognosis of C2 and C3 (**Figures 7C, D**). These results suggest that our risk model can distinguish patients with different risks and provide reference for clinical prognosis prediction of patients.

### Correlation between risk model and immunity

As shown in **Supplementary Figures 3A, B**, tumor tissue infiltration levels of aDCs, CD8+ T cells, macrophages, T helper cells, Tfh, Th2 cells, and TIL were higher in high-risk patients with COAD in TCGA than in low-risk patients. In COAD patients with GEO, the infiltration levels of aDCs, B cells, CD8+ T cells, DCs, macrophages, neutrophils, T helper cells, Tfh, Th2 cells, and TIL in tumor tissues of high-risk patients were higher than those of low-risk patients, suggesting that our risk model could reflect the immune infiltration of patients. In addition, **Supplementary Figures 3C, D** respectively show the relationship between different risk patients and immune function in TCGA and GEO, and the results suggest that high-risk patients are related to the activation of multiple immune functions. These results suggest that our risk model is associated with immune cell infiltration and immune function, suggesting that MMRG was associated with tumor immunity.

### Correlation of CPT2 expression and tumor immune infiltration in clinical specimens

**Figure 8:**
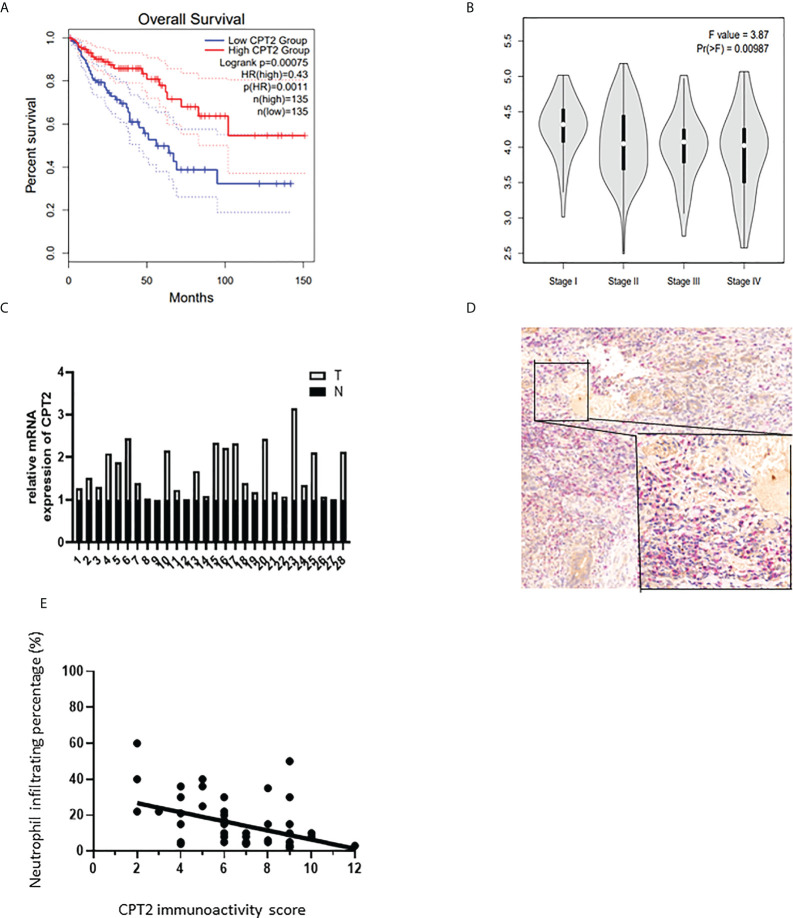
Correlation of CPT2 expression and immune infiltration in human specimens. **(A, B)** Correlation of CPT2 expression with Kaplan–Meier curves and clinical stages in COAD patients. **(C)** Relative mRNA expression of CPT2 in 28 paired human specimens, N normal vs. T tumor. **(D)** Representative multiplexed IHC image of tissue area (2 mm × 1.5 mm) with an enlarged image for COAD human specimens, showing staining of CPT2 (brown) and MPO (red). **(E)** Spearman correlation analyses suggested that the expression of CPT2 negatively correlated with that of MPO (Spearman r = −0.42, p < 0.01). Scale bars are 20 and 200 μm in **(D)**.

To identify the correlations between MMRGs and clinical parameters, we investigated the association between the expression levels of the 28 differentially expressed genes (DEGs) and their prognosis and clinical staging. We found that the expression levels of four DEGs, namely, ACAA2 (p < 0.05), ADH6 (p < 0.05), CPT2 (p < 0.01), and PPARGC1A (p < 0.01), had survival significance (**Figure 8A**; **Supplementary Figure 4**). Among the four DEGs, only ADH6 (p < 0.01) and CPT2 (p < 0.01) were negatively related to the clinical stage and met statistical significance (**Figure 8B**; **Supplementary Figure 5**). Therefore, we chose ADH6 and CPT2 as potential targets for further study.

As shown in **Figure 3A**, we revealed that CPT2 was significantly correlated with immune infiltration, including neutrophils (p < 0.05) and NK cells (p < 0.01). Correlation analyses using data from the TIMER2 database showed that CPT2 was correlated with the infiltration levels of CD8+ T cells (r = 0.262, p < 0.001), CD4+ T cells (r = 0.111, p < 0.05), and neutrophils (r = 0.174, p < 0.001) (**Supplementary Figure 6**). We used 28 colorectal cancer and paired adjacent normal tissues and found that CPT2 expression was downregulated in tumor (p = 0.0027) (**Figure 8C**). To evaluate the correlation of CPT2 and neutrophil infiltration in human CRC tissues, we performed immunohistochemical staining for CPT2 and MPO on tissue microarrays. A representative picture is shown in **Figure 8D**. However, Spearman correlation analyses suggested that the expression of CPT2 negatively correlated with MPO (Spearman r = −0.42, p < 0.01), which is contrary to the results of bioinformatics (**Figure 8E**).

### CPT2 modulates migration and apoptosis of neutrophils

**Figure 9:**
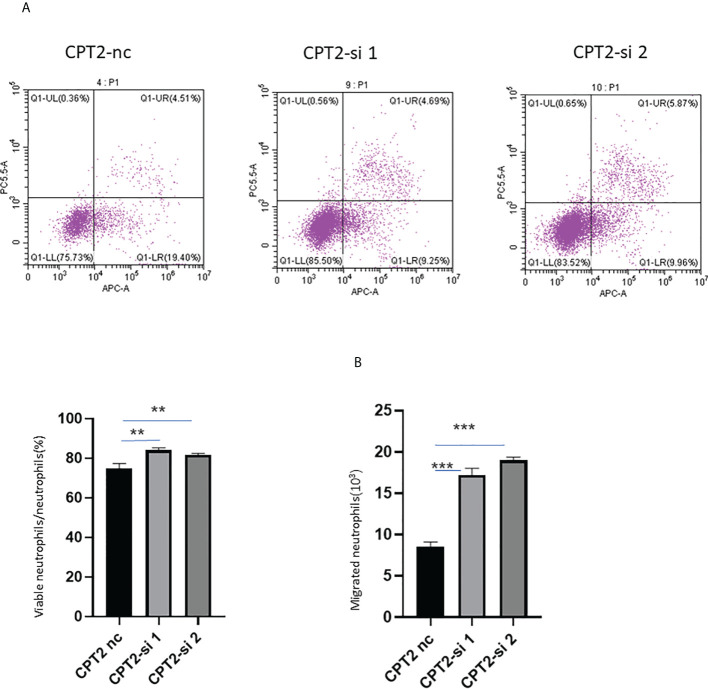
CPT2 modulates migration and apoptosis of neutrophils. **(A)** Migration of neutrophils toward CM from CPT2-si and CPT2-nc RKO cells was evaluated using *in vitro* Transwell migration assay in triplicate (one-way ANOVA test). **(B)** Tumor cell-derived conditioned medium from RKO cells with different expressions of CPT2 alter neutrophil survival. Data are presented as means ± SEM. **p < 0.01; ***p < 0.001.

The role of CPT2 in the accumulation of neutrophils was tested by assessing whether the conditioned medium (CM) from CRC cells with different expressions of CPT2 could alter neutrophil apoptosis or migration *in vitro*. The knockdown efficiency of small interfering RNA was detected by RT-qPCR (**Supplementary Figure 7**). There was increased neutrophil migration and deduced neutrophil apoptosis toward the CM derived from si-CPT2 cells (**Figures 9A, B**). Together, these findings are consistent with the conclusion that CPT2 is a key mediator of neutrophil recruitment in CRC.

## Discussion

Analysis of the MMRGs in the COAD, suggested that they were involved in different processes and associated with tumor immunity. By using differentially expressed genes, COAD patients could be divided into two subtypes, and the risk model could well predict the prognosis of patients. All these imply that MMRGs have potential clinical value in predicting patients' prognoses as well as guiding relevant immunotherapy.

Mitochondria are the main energy providers of the body and are involved in many biological processes, including cell homeostasis, energy growth, and apoptosis. The catabolism process of glucose, fatty acids, and amino acids is ultimately completed in the mitochondria of the cell. Abnormal mitochondrial genes and changes in mitochondrial metabolic pathways can affect the expression of cancer-related genes *in vivo*, thus promoting tumor development and immune escape (24). Shi et al. found that mitochondrial dysfunction can enhance the resistance of COAD to radiotherapy, thus promoting tumor progression (25). Cheng et al. found that metastatic COAD can change the mitochondrial metabolic pattern to enable tumor survival under low energy conditions, thus supporting tumor growth (26). Oxidative phosphorylation of mitochondria has become an important field of tumor therapy (27, 28). COAD stem cells maintain their survival by oxidative phosphorylation and glycolysis and promote tumor cell resistance to 5-Fu and antimycin A through oxidative phosphorylation (29). In addition, inhibition of oxidative phosphorylation enhances the sensitivity of COAD tumor cells to drug therapy (30). We used MMRGs to divide COAD into two subtypes, and the prognosis of patients with different MMRG subtypes was different. Meanwhile, the risk model constructed using MMRGs can well predict the prognosis of patients. These results suggest that mitochondrial dysfunction and changes in energy metabolism are important features of cancer, especially COAD, as well as important biological targets for COAD treatment, and patients’ mitochondrial metabolism patterns affect their clinical prognosis.

Most of the genes associated with mitochondrial energy metabolism used to construct risk models were involved in the growth and progression of multiple tumors. In melanoma, ACOX1-mediated fatty acid oxidation is involved in tumor resistance to BRAF/MEK inhibitors (31). In oral squamous cell carcinoma, ACOX1 boosts tumor cell viability (32). In COAD, downregulation of CPT2 promotes tumor resistance to oxaliplatin, while in ovarian cancer, downregulation of CPT2 promotes tumor growth and metastasis (33, 34). ADH6 is downregulated in hepatocellular carcinoma and is an important prognostic marker of pancreatic cancer (35, 36). PPARGC1A can promote tumor metastasis by promoting oxidative phosphorylation, while PPARGC1B can promote the proliferation of HER2-overexpressed breast cancer cells (37, 38). ACADL can inhibit the proliferation of hepatocellular carcinoma tumor cells through Hippo/YAP signaling (39). ALDH4A1 is downregulated in lung cancer and is an important biomarker for differentiating lung cancer tumor tissue from normal tissue (22). These studies indicated that the genes used to construct risk models were associated with tumors, and therefore, our risk model was a tumor-associated risk model.

The tumor immune microenvironment plays an important role in tumor immunotherapy (40). Infiltration of specific subsets of functional immune cells within the tumor can influence the prognosis and risk of postoperative recurrence in patients with COAD (41). Mitochondria can influence immune monitoring through internal and external mechanisms of cancer cells (11). In addition, immune cells in the tumor microenvironment can also influence the changes of mitochondrial metabolic patterns in the tumor, thus stimulating the growth of cancer cells and inhibiting cell apoptosis (42). In our study, we found that MMRG was associated with infiltration of various immune cells and immune function. MMRG-based COAD typing and related risk models were found to be correlated with tumor immunity in immune correlation analysis. Therefore, MMRG not only participates in the construction of energy metabolism mode of tumor cells, enabling tumor cells to survive in the environment of oxidative stress and nutrient deficiency, but also participates in the process of immunity in tumor tissues and further promotes tumor progress through the interaction between tumor cells and immune cells.

Our study showed that MMRG-based COAD patient typing could well distinguish patients with different prognoses, and the MMRG-based risk model could well predict the clinical prognosis of patients. CPT2 exhibited potential clinical predictive and prognostic value based on a series of bioinformatic analyses. We primarily reveal that CPT2 was related to tumor immune infiltration and may act as a valuable biomarker for COAD immunotherapy. However, there are still two shortcomings. Firstly, a large number of clinical samples are still required to further validate the clinical value of typing and risk models. Secondly, insights into the molecular mechanisms of CPT2 in tumorigenesis should be elucidated by further laboratory work.

## Conclusions

Using TCGA database, we found 28 differentially expressed MMRGs and found 188 MMRGs involved in different biological processes in normal colon and tumor tissues. In addition, MMRG-based clinical subtype analysis was able to distinguish COAD patients with different clinical outcomes, while the associated risk model was able to predict the prognosis of patients with COAD. Finally, we found that not only the MMRG gene but also clinical classification and risk model based on MMRG were correlated with tumor tissue immune cell infiltration and related immune function of tumor patients. We preliminarily recognize CPT2 as a potential tumor-suppressor gene and is associated with a state of neutrophil infiltration.

## Data availability statement

The original contributions presented in the study are included in the article/**Supplementary Material**. Further inquiries can be directed to the corresponding authors.

## Ethics statement

This study was reviewed and approved by Ruijin Hospital, Shanghai Jiaotong University school of medicine. The patients/participants provided their written informed consent to participate in this study.

## Author contributions

The article was written by ZC. JL contributed equally to this work. WC and ZY have provided guidance to the manuscript preparation. All authors contributed to the article and approved the submitted version.

## Funding

This work was supported by the Shanghai Municipal Science and Technology Commission (19441905400), Shanghai Jiaotong University (YG2019ZDA15), and Shanghai Municipal Commission of Health and Family Planning (No. 2017-239).

## Acknowledgments

We thank all the authors who contributed to this topic. Thanks are also given to TCGA and GEO databases for providing data.

## Conflict of interest

The authors declare that the research was conducted in the absence of any commercial or financial relationships that could be construed as a potential conflict of interest.

## Publisher’s note

All claims expressed in this article are solely those of the authors and do not necessarily represent those of their affiliated organizations, or those of the publisher, the editors and the reviewers. Any product that may be evaluated in this article, or claim that may be made by its manufacturer, is not guaranteed or endorsed by the publisher.
