# Supplementary material for: An integrated bioinformatic investigation of mitochondrial energy metabolism genes in colon adenocarcinoma followed by preliminary validation of CPT2 in tumor immune infiltration
Source: Front Immunol. 2022 Sep 13;13:959967. doi: 10.3389/fimmu.2022.959967 (PMC9513322; doi:10.3389/fimmu.2022.959967)
Supplement: Supplementary file 1 [file DataSheet_1.pdf]

Supplementary Information for

Mitochondrial Energy Metabolism Pattern and Tumor Microenvironment Characterization in Colon Adenocarcinoma

Zichao Cao [1], Jianwei Lin[1], Zheyu Yang[1], Wei Cai[1*]

[1]Department of General Surgery, Ruijin Hospital, Shanghai Jiao Tong University School of Medicine. Shanghai, Shanghai, China

Corresponding Author:
Wei Cai[1]
Email address: caiwei@shsmu.edu.cn

Keywords: mitochondrion 1, energy metabolism 2, colon adenocarcinoma 3, riskscore4, microenvironment5.

This file includes:

Figure S1-S7

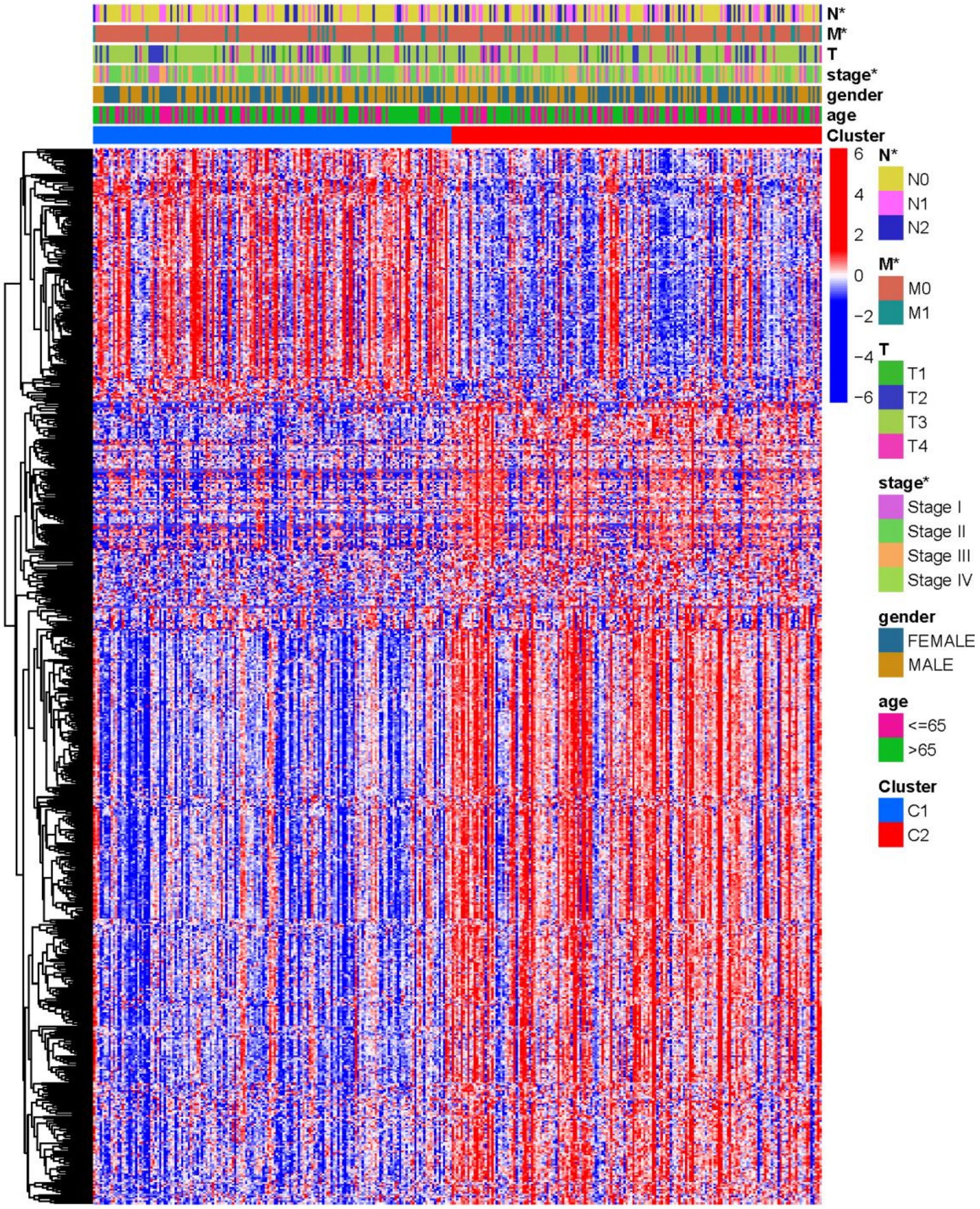

Supplementary Figure S1. Heatmap of genes differentially expressed between subtypes differentiated by MMRG, with blue representing down-regulation and red representing up-regulation.

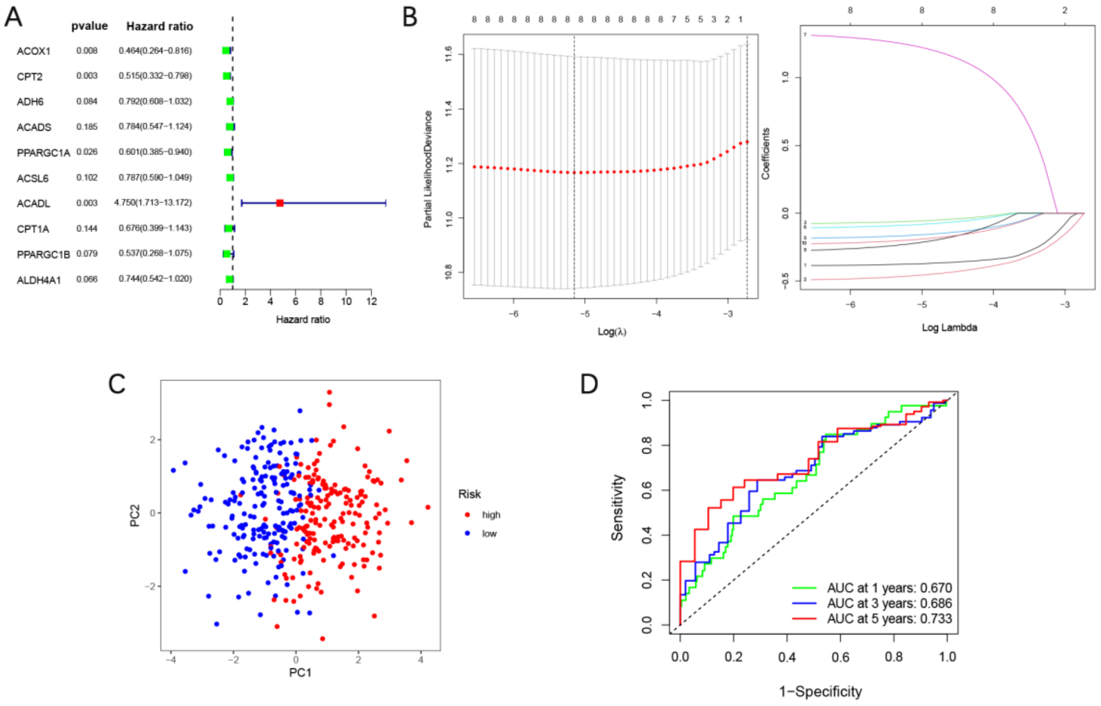

Supplementary Figure S2. Risk score model construction. (A) Univariate cox regression analysis of MMRGs. Green is a prognostic protective gene; red is a prognostic risk gene. (B) Distribution of Lasso analysis for ten genes identified by univariate cox regression analysis and coefficients for eleven genes analyzed by Lasso. Two vertical lines represent lambda.min and lambda.lse. (C) PCA stratified analysis in TCGA database. (D) ROC for risk score model in TCGA database. PCA,Principal Component Analysis; ROC, Receiver Operating Characteristic Curve.

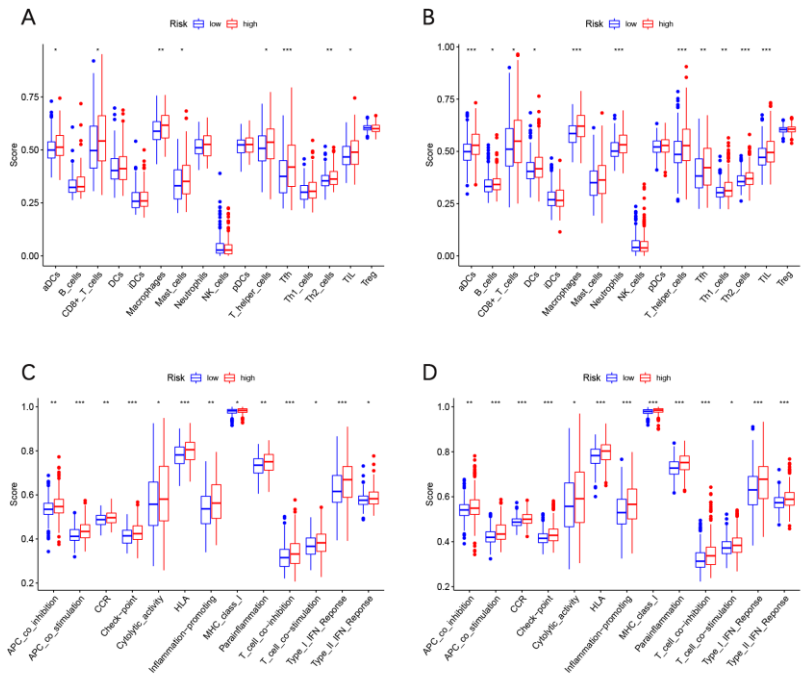

Supplementary Figure S3. Correlation of the risk score with immune cells and immune function. (A, B) Comparison of the enrichment scores of 16 types of immune cells between low- (blue box) and high-risk (red box) groups in the TCGA cohort and GEO cohort. (C, D) Comparison of the enrichment scores of 13 immune-related functions between low- (blue box) and high-risk (red box) groups in the TCGA cohort and GEO cohort.

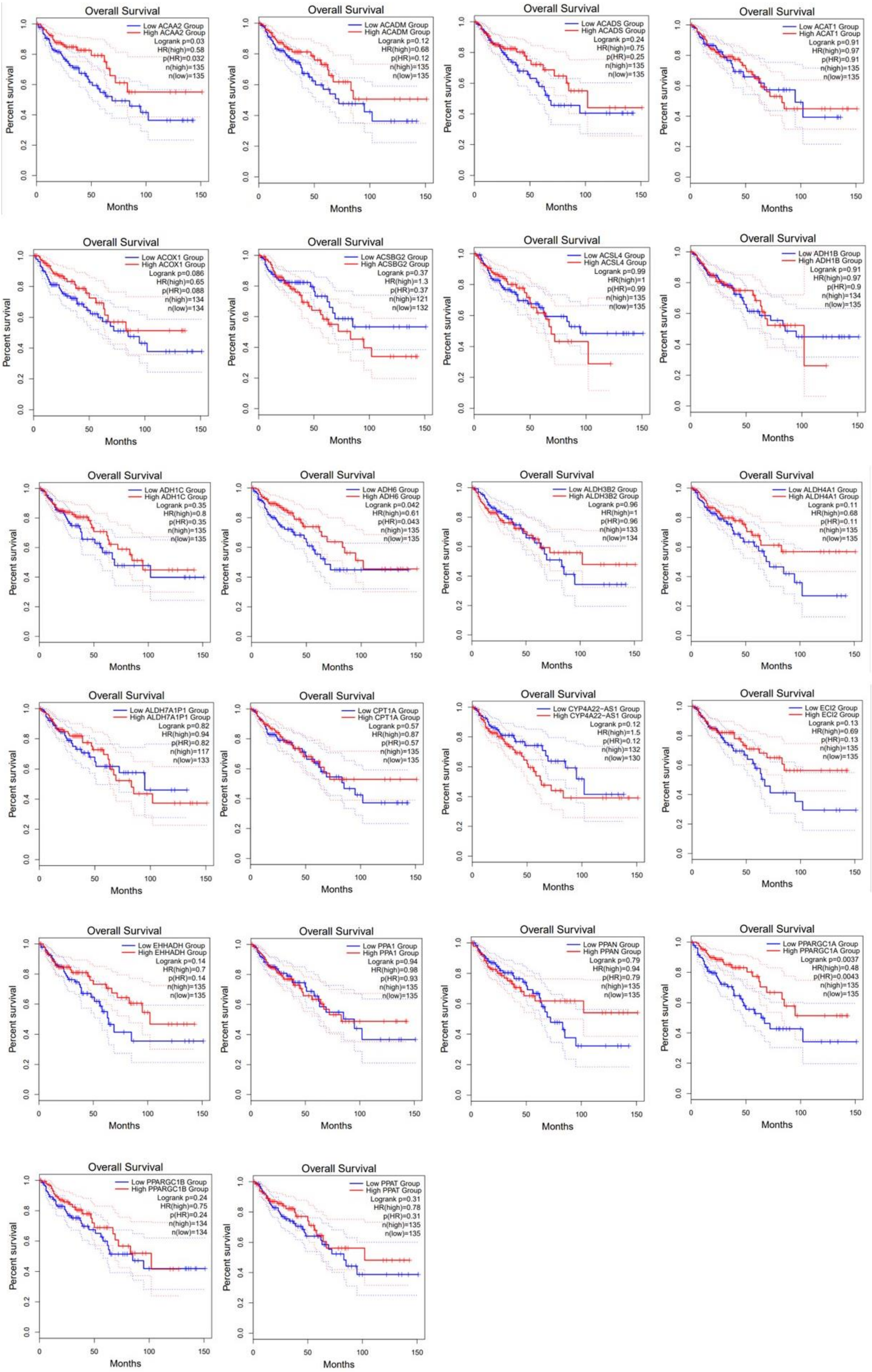

Supplementary Figure S4: Kaplan–Meier curves suggested that patients with downregulated ADH6, CPT2, PPARGC1A and ACAA2 had a longer overall survival (OS) time.

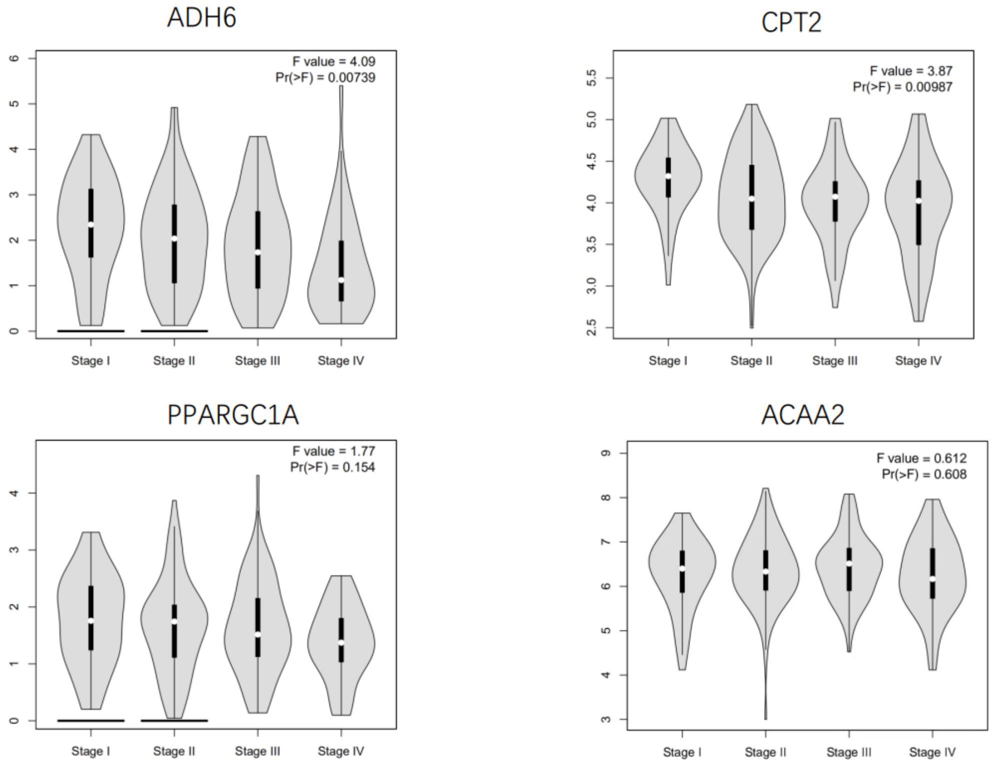

Supplementary Figure S5: Expressions of survival-related differentially expressed MMRGs (ADH6, CPT2, PPARGC1A, and ACAA2) between different stages of colon cancers.

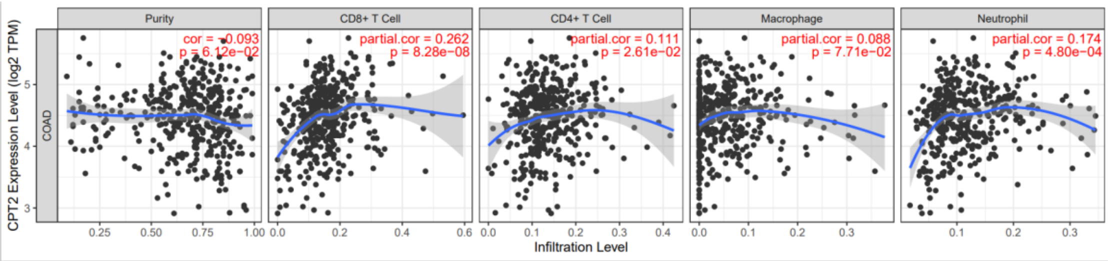

Supplementary Figure S6: The expression of CPT2 was positively related to the infiltration of CD8+ T cells (r=0.262, p< 0.001), CD4+ T cells (r=0.111, p< 0.05), and neutrophils (r=0.174, p< 0.001) by Spearman relation analysis.

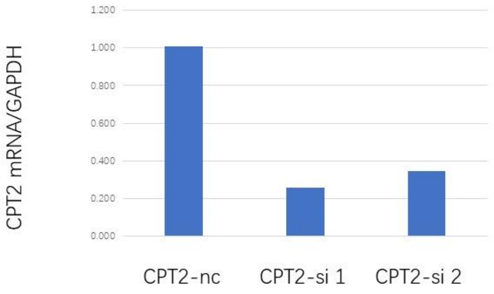

Supplementary Figure S7: The knockdown efficiency of CPT2 was detected by RT-qPCR.
